# HCN channels contribute to the sensitivity of intravenous anesthetics in developmental mice

**DOI:** 10.18632/oncotarget.24408

**Published:** 2018-02-05

**Authors:** Jie Gao, Zhiqiang Hu, Liwei Shi, Na Li, Yeling Ouyang, Shaofang Shu, Shanglong Yao, Xiangdong Chen

**Affiliations:** ^1^ Department of Anesthesiology, Union Hospital, Tongji Medical College, Huazhong University of Science and Technology, Wuhan 430022, China; ^2^ Institute of Anesthesiology and Critical Care Medicine, Union Hospital, Tongji Medical College, Huazhong University of Science and Technology, Wuhan 430022, China

**Keywords:** HCN channels, intravenous anesthetics, development, sensitivity

## Abstract

It is widely accepted that the induction dose of anesthetics is higher in infants than in adults, although the relevant molecular mechanism remains elusive. We previously showed neuronal hyperpolarization-activated, cyclic nucleotide-gated (HCN) channels contribute to hypnotic actions of propofol and ketamine. Interestingly, the expression of HCN channels in neocortex significantly changes during postnatal periods. Thus, we postulated that changes in HCN channels expression might contribute to sensitivity to intravenous anesthetics. Here we showed the EC_50_ for propofol- and ketamine-induced loss-of-righting reflex (LORR) was significantly lower for P35 than for P14 mice. Cerebrospinal fluid concentrations of propofol and ketamine were significantly higher in P14 mice than in P35 mice, with similar propofol- and ketamine-induced anesthesia at the LORR EC_50_. Western blotting indicated that the expression of HCN channels in neocortex changed significantly from P14 to P35 mice. In addition, the amplitude of HCN currents in the neocortical layer 5 pyramidal neurons and the inhibition of propofol and ketamine on HCN currents dramatically increased with development. Logistic regression analysis indicated that the changes of HCN channels were correlated with the age-related differences of propofol- and ketamine-induced anesthesia. These data reveal that the change of HCN channels expression with postnatal development may contribute to sensitivity to the hypnotic actions of propofol and ketamine in mice.

## INTRODUCTION

It is well established that the induction dose of anesthetics is influenced by age [1–3]. This age-related phenomenon has been observed in animals and humans in previous studies. Earlier works demonstrated that the dose requirement for induction with propofol was higher in infants and children than in adults in clinical practice [1, 2], and these differences were substantial during the important developmental stages from infants to children [2]. A study from 1980 also reported that adult pigtail monkeys were significantly more sensitive to the hypnotic effects of ketamine than neonatal and young pigtail monkeys [4]. Since millions of infants and children undergo anesthesia around the world every year, an accurate understanding of these age-related differences is very important for clinical practice and basic research.

Previous studies explained this age-related sensitivity to anesthetics based on the different pharmacokinetics parameters in patients of different ages [2, 3], as infants and children have higher cardiac output relative to body weight and are considered to exhibit lower concentration of anesthetics in the blood perfusing the brain compared to adults [2, 4]. However, the age-related changes were significantly different between anesthesia with propofol or with thiopental, another intravenous anesthetic, even though thiopental and propofol have similar pharmacokinetics parameters [1, 2], indicating that differences in pharmacokinetics parameters could not fully explain the age-related differences in the induction dose of anesthetics [2–4].

Other authors favored the idea that changes in pharmacodynamics during different developmental periods contribute to age-related sensitivity to the hypnotic actions of anesthetics [1]. A clinical trial using pharmacodynamics models and analysis found that the increased induction dose of propofol correlated with the different pharmacodynamics parameters in patients in different developmental stages [1–4], suggesting that pharmacodynamics plays an important role in age-related sensitivity to anesthetics. Receptors and ion channels, such as N-methyl-D-aspartic acid (NMDA) receptors, γ-aminobutyric acid type A (GABAA) receptors and hyperpolarization-activated, cyclic nucleotide-gated (HCN) channels [5–8], are believed to be involved in the pharmacodynamics of anesthetics.

It is now clear that HCN channels are involved in the hypnotic effects of intravenous anesthetics [9–15]. Previous studies have demonstrated that general anesthetics, such as propofol and ketamine, produce anesthetic activities through the inhibition of HCN channels and that HCN1 knockout mice are significantly less sensitive to these anesthetics [9, 10, 15].

HCN (1-4) channels are the products of a 4-member hyperpolarization-activated cationic gene family [5, 10, 16]. Among HCN channel subtypes, the HCN1 subtype is strongly expressed in the cerebral cortex and regulates the excitability of cortical neurons [9, 10, 15], which is a feature of general anesthetic-induced hypnosis [15, 16]. Previous works have demonstrated that cortical excitability is principally controlled by HCN1 channels in the layer 5 pyramidal neurons [17–19] and that an age-related increase in HCN1 channels enhanced these actions [17]. Recent studies indicated that the expression of HCN1 channels and the HCN current showed nearly 2-fold changes in the neocortical dendritic tree of layer 5 pyramidal neurons during the first postnatal month in mice [17–20]. Thus, we postulated that HCN channels contribute to higher sensitivity to intravenous anesthetics with increasing age.

In the present study, we combined behavioral measurement, molecular biological methods, high-performance chromatography and whole-cell recording to identify the relationship between the expression level of HCN channels and sensitivity to anesthetics in mice.

## RESULTS

### The LORR EC_50_ values of propofol and ketamine anesthesia are decreased with the development of mice

**Figure 1:**
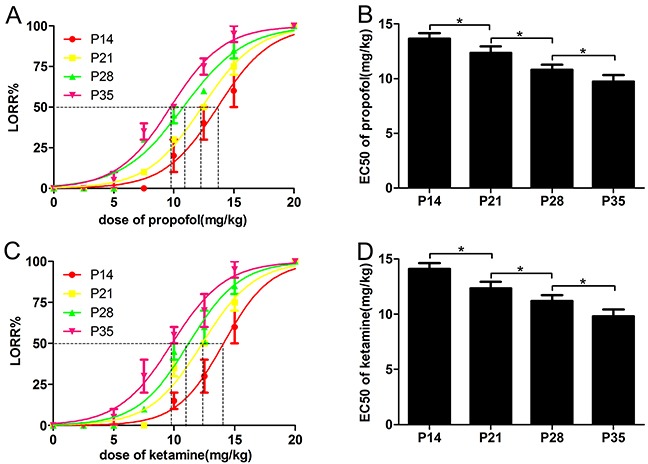
The EC50 of the loss-of-righting reflex (LORR) induced by propofol and ketamine was reduced with the development of mice **(A** and **B)** Mice were injected with increasing doses of propofol (2.5-20 mg/kg, IV), and the percentage of P14, P21, P28 and P35 mice that failed to right themselves (LORR) was determined as a measure of hypnosis. P35 mice were more sensitive to the hypnotic effect of propofol than P14 mice, indicated by the decreased EC_50_ for propofol-induced LORR (n=10, F=75.39, ^*^P<0.05 using one-way ANOVA). **(C** and **D)** Mice were injected with increasing doses of ketamine (2.5-20 mg/kg, IV), and the LORR percentage of P14, P21, and P28 and P35 mice was determined as a measure of hypnosis. P35 mice were more sensitive to the hypnotic effect of ketamine than P14 mice, as indicated by the decreased EC_50_ for ketamine-induced LORR (n=10, F=76.62, ^*^P<0.05 using one-way ANOVA).

To investigate different responses to intravenous anesthetics in mice during different developmental periods, we used LORR analysis to examine the hypnotic actions of propofol and ketamine in developmental mice [9, 21]. The LORR EC_50_ of propofol decreased with the development of mice (Figure 1A and 1B, EC_50_: 13.66 ± 0.20 mg/kg for P14 mice, 12.35 ± 0.19 mg/kg for P21 mice, 10.80 ± 0.17 mg/kg for P28 mice and 9.73 ± 0.20 mg/kg for P35 mice, n=10, F=167.9, *P*<0.001 using one-way ANOVA). Similar to propofol, the LORR EC_50_ of ketamine (Figure 1C and 1D) showed a similar trend (EC_50_: 14.09 ± 0.23 mg/kg for P14 mice, 12.33 ± 0.20 mg/kg for P21 mice, 11.21 ± 0.20 mg/kg for P28 mice, and 9.82 ± 0.20 mg/kg for P35 mice, n=10, F=151.6, *P*<0.001 using one-way ANOVA). Since the sex differences in sodium pentobarbital anesthesia have been reported in previous study [22], we also analyzed the data and found there was no difference between male and female mice ((propofol: 13.78 ± 0.20 mg/kg *vs.* 13.12 ± 0.36 mg/kg for P14 mice, 12.25 ± 0.29 mg/kg vs. 12.55 ± 0.14 mg/kg for P21 mice, 10.56 ± 0.37 mg/kg *vs*. 10.77 ± 0.56mg/kg for P28 mice and 9.43 ± 0.20 mg/kg *vs.* 9.68 ± 0.32 mg/kg for P35 mice, n=5, *P*>0.05; ketamine: 13.89 ± 0.16 mg/kg *vs.* 14.04 ± 0.28mg/kg for P14 mice, 12.43 ± 0.26 mg/kg *vs.* 12.56 ± 0.35 mg/kg for P21 mice, 11.45 ± 0.45 mg/kg *vs.* 11.28 ± 0.35mg/kg for P28 mice, and 9.88 ± 0.12 mg/kg *vs.* 9.78 ± 0.46 mg/kg for P35 mice, n=5, *P*>0.05). These data indicated that the anesthetic effects of propofol and ketamine on behavior involving the righting reflex were affected by the developmental age of mice.

### With increasing age, decreased CSF concentrations of propofol and ketamine are required to produce the same anesthetic actions

**Figure 2:**
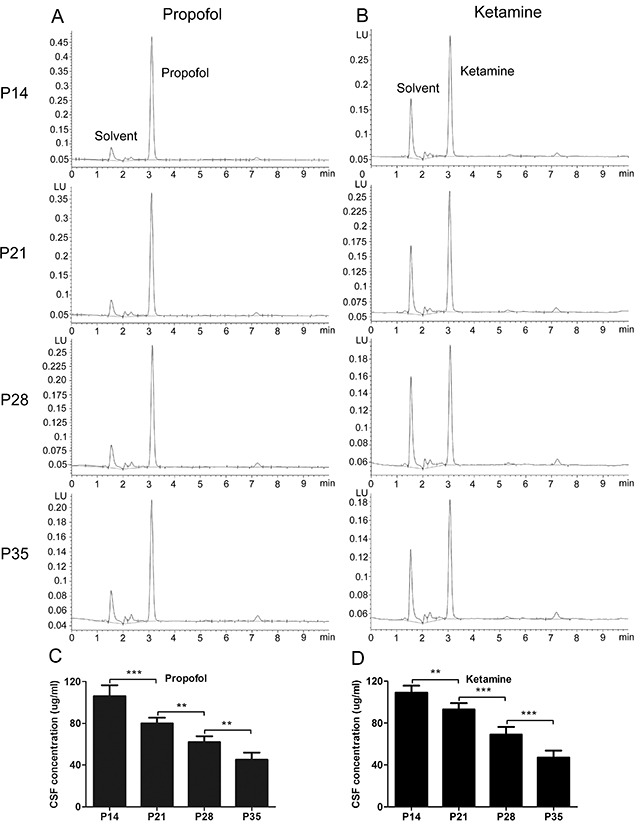
Cerebrospinal fluid (CSF) concentrations of propofol and ketamine needed to produce the same LORR decreased with increasing age of mice **(A)** Representative high-performance liquid chromatography (HPLC) revealed the CSF concentration of propofol after injection. **(C)** Mice were injected with the LORR EC_50_ of propofol (IV), calculated from behavioral tests, and the CSF concentration of propofol was immediately detected after injection. P14 mice were less sensitive to the hypnotic effects of propofol, as indicated by the increased CSF concentration of propofol (n=5, F=62.50, ^**^P<0.01, ^***^P<0.001 using one-way ANOVA). **(B)** Representative HPLC revealed the CSF concentration of ketamine. **(D)** Mice were injected with the EC_50_ of ketamine (IV), calculated from behavioral tests, and the CSF concentration of ketamine was immediately detected after injection. P14 mice were less sensitive to the hypnotic effects of ketamine, as indicated by the increased CSF concentration of ketamine (n=5, F=81.39, ^**^P<0.01, ^***^P<0.001 using one-way ANOVA).

Studies have proposed that in animals, the sensitivity to intravenous anesthetics changes with the developmental age, reflecting pharmacokinetic differences rather than pharmacodynamics changes, while others have suggested that age-related physiological changes in pharmacodynamics are important in addition to the well-known differences in pharmacokinetics [1–3, 23]. To differentiate the pharmacokinetic and pharmacodynamic changes reflecting differences in the hypnotic actions of intravenous anesthetics with developmental age, we measured the CSF concentrations of anesthetics to reflect the brain’s sensitivity to these drugs. To examine the CSF concentrations of propofol and ketamine, HPLC was used to rapidly detect the concentrations of propofol and ketamine in CSF after a single bolus injection with a dose of LORR EC_50_ calculated from behavioral tests (Figure 2A and 2B). The results showed that the CSF concentration of propofol gradually decreased from P14 to P35 in mice (Figure 2C, CSF concentration of propofol: 106 ± 10.43 μg/ml for P14 mice, 80 ± 5.46 μg/ml for P21 mice, 62 ± 5.53 μg/ml for P28 mice, and 45 ± 6.98 μg/ml for P35 mice, n=5, F=62.50, *P*<0.001 using one-way ANOVA). Similar results were observed for ketamine (Figure 2D, CSF concentration of ketamine: 109 ± 6.74 μg/ml for P14 mice, 93 ± 6.07 μg/ml for P21 mice, 69 ± 7.37 μg/ml P28 mice, and 47 ± 6.72 μg/ml for P35 mice, n=5, F=81.39, *P*<0.001 using one-way ANOVA). These results indicated that the CSF concentrations of propofol and ketamine that produced the same LORR decreased with the developmental age of mice, implying that the sensitivity to propofol and ketamine anesthesia is indeed different during different developmental stages and that the sensitivity to intravenous anesthetics is correlated with age-related differences in pharmacodynamics [1–3].

### Expression level of HCN1 channels is closely correlated with the sensitivity to propofol- and ketamine-induced anesthesia

**Figure 3:**
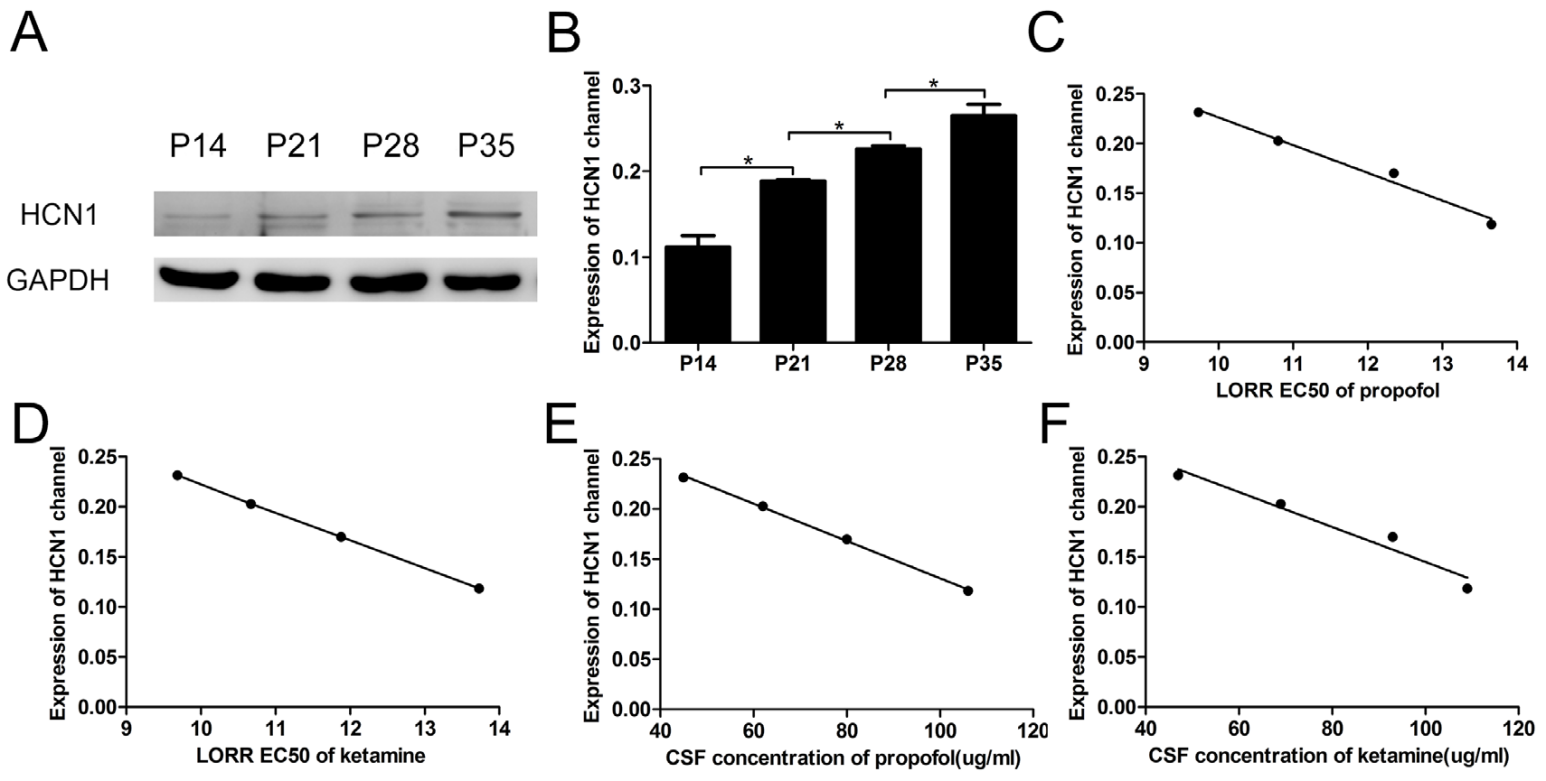
The LORR EC50 and CSF concentrations of propofol and ketamine correlated with the expression of the HCN1 channel in the cortex **(A)** Representative Western blots show HCN1 protein expression in the cortex of P14, P21, P28 and P35 mice. **(B)** Quantitative analysis of the HCN1 channel protein (relative to GAPDH). Values are expressed as the means ± SEM. These results indicated that the expression of HCN1 channels increased with the development of mice (n=5, F=139.4, ^*^P<0.05, using one-way ANOVA). **(C** and **D)** Relationship between the expression of the HCN1 channel in the cortex and the LORR EC_50_ of propofol and ketamine using logistic regression. These data show that the LORR EC_50_ values of propofol and ketamine were closely correlated with the expression of the HCN1 channel at different developmental periods in mice (propofol: R^2^=0.98, 95% CI=-0.99 to −0.52, *P*<0.05, ketamine: R^2^=0.98, 95% CI=-0.99 to −0.56, *P*<0.05 using logistic regression). **(E** and **F)** Relationship between HCN1 channel expression in the cortex and the CSF concentrations of propofol and ketamine using logistic regression. These data show that the CSF concentrations of both propofol and ketamine were closely correlated with the expression of the HCN1 channel at different developmental periods in mice (CSF concentration of propofol and HCN1 channel: R^2^=0.99, 95% CI=-1.00 to −0.96, *P*<0.001; CSF concentration of ketamine and HCN1 channel: R^2^=0.95, 95% CI=-0.99 to −0.25, *P*<0.05 using logistic regression).

There are four different HCN subunits expressed to some degree in the mammalian central nervous system (CNS) [24, 25]. Among these subunits, HCN1 channels are strongly inhibited by anesthetics [9–11]. We examined the expression of HCN1 channels in the mouse cortex and observed that the expression increased dramatically by approximately 92% from P14 to P35 (Figure 3A and 3B, n=5, F=139.4, *P*<0.001 using one-way ANOVA).

We also analyzed the relationship between the expression of HCN1 channels and the LORR EC_50_ of propofol and ketamine. Using logistic regression, the expression of HCN1 channels showed a close correlation with the LORR EC_50_ in each group (Figure 3C and 3D, propofol: R^2^=0.98, 95% CI=-0.99 to −0.52, *P*<0.05, and ketamine: R^2^=0.99, 95% CI=-0.99 to −0.56, *P*<0.05, using logistic regression). In addition, a clear linear correlation between the expression of HCN1 channels and the CSF concentrations of propofol and ketamine after the administration of LORR EC_50_ was also observed (Figure 3E and 3F, propofol: R^2^=0.99, 95% CI=-1 to −0.96, *P*<0.001, and ketamine: R^2^=0.95, 95% CI=-0.99 to −0.25, *P*<0.05 using logistic regression). These results establish a mechanism for the increased sensitivity to propofol and ketamine anesthesia with increasing age, which could reflect the expression changes of HCN1 channels during development.

### The HCN current in neocortical pyramidal neurons significantly increases with development in mice

**Figure 4:**
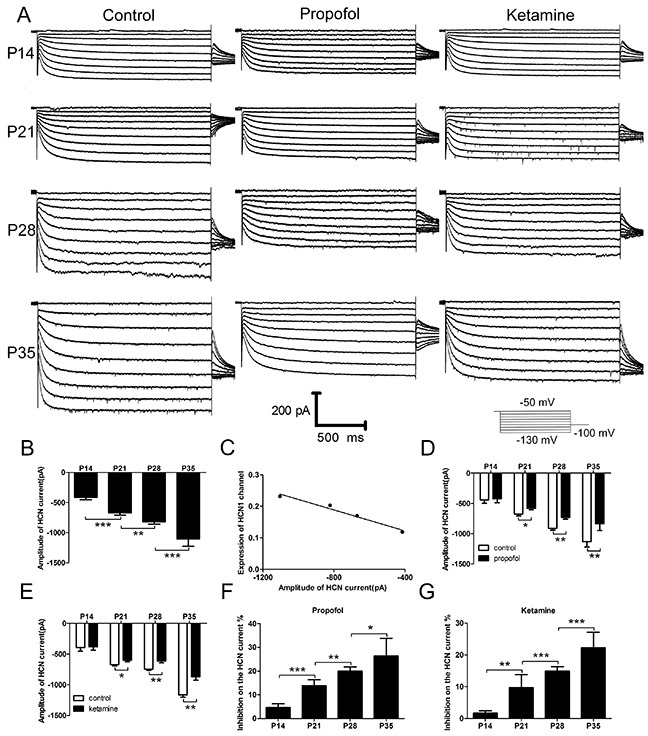
HCN current and the effects of propofol and ketamine on the HCN current in cortical pyramidal neurons increased with the development of mice **(A)** Sample voltage clamp recordings of HCN current in cortical pyramidal neurons from mice at different ages evoked by hyperpolarizing voltage steps from −50 to −130 mV under control conditions (left) or during exposure to propofol (5 μM, middle) and ketamine (20 μM, right). **(B)** Averaged means ± SEM depict the HCN amplitude (at −130 mV) in cortical pyramidal neurons from mice of different development periods. These data showed that the HCN current increased with the development of mice (n=20, F=79.49, ^**^P<0.01, ^***^P<0.001 using one-way ANOVA). **(C)** Relationship between expression of the HCN1 channel in the cortex and the amplitude of the HCN current using logistic regression. These data show that the amplitude of HCN current was closely correlated with the expression of HCN1 channels at different developmental periods in mice (R^2^=0.97, 95% CI=-0.99 to −0.43, *P*<0.05 by logistic regression). **(D** and **E)** The effects of propofol (5 μM) and ketamine (20 μM) on maximal current amplitude were determined in cortical pyramidal neurons. These data show that there were little effects of propofol and ketamine on the HCN current in P14 mice but significant effects in P21, P28, and P35 mice (propofol: n=10, F=67.2, ^*^P<0.05, ^**^P<0.01;ketamine: n=10, F=70.2, ^*^P<0.05, ^**^P<0.01 using two-way ANOVA). **(F** and **G)** (I _control_-I _anesthetic_) / I _control_ was used to determine the inhibition% ratio from C and D. These results revealed that the inhibition of propofol and ketamine on HCN current increased with the development of mice (propofol: n=10, F=55.94, ^*^P<0.05, ^**^P<0.01, ^***^P<0.001, ketamine: n=10, F=41.53, ^**^P<0.01, ^***^P<0.001, using one-way ANOVA).

Because layer 5 pyramidal neurons play a critical role in the hypnotic actions of anesthetics in the cortex, we used whole-cell recording to identify changes of the HCN current in cortical pyramidal neurons [19, 20]. The results showed that the HCN current in cortical pyramidal neurons steadily increased with increasing age (Figure 4A left), and the maximal HCN current amplitude in P35 mice was nearly 2-fold that of P14 mice (Figure 4B, the maximal amplitude of HCN current: −415.09 ± 36.89 pA for P14 mice, −670.01 ± 39.28 pA for P21 mice, −821.77 ± 37.01 pA for P28 mice, and −1103.45 ± 124.35 pA for P35 mice, n=20, F=79.49, *P*<0.001 using one-way ANOVA), consistent with the results of previous studies [17–19, 25]. Moreover, in the present study, a clear linear correlation existed between the expression of HCN1 channels and the maximal amplitude of HCN current (Figure 4C, R^2^=0.97, 95% CI=-0.99 to −0.43, *P*<0.05 using logistic regression). These findings prompted an investigation into whether there are different effects of propofol and ketamine on the HCN current.

### Different effects of propofol and ketamine on HCN current in neocortical pyramidal neurons in mice during different developmental periods

HCN channels contribute to the anesthetic activities of propofol and ketamine [9–12]. Therefore, we next assessed whether there were altered effects of propofol and ketamine on HCN current with the development of mice. We established whole-cell patch-clamp brain slices in mice to characterize the HCN current and determine its modulation by propofol and ketamine (Figure 4A, middle and right). The results showed that both propofol (5 μM, for ~5 min) and ketamine (20 μM, for ~5 min) significantly inhibited the HCN current in P21, P28 and P35 mice (Figure 4D and 4F, inhibition of propofol on maximal current: −675.15 ± 29.51 pA to −580.33 ± 22.93 (9.86% ± 4.08%) for P21mice, −909.51 ± 31.13 pA to −727.99 ± 31.09 pA (14.95% ± 1.30%) for P28 mice, and −1133.32 ± 85.43 pA to −832.43 ± 114.68 pA (22.22% ± 4.89%) for P35 mice, n=10, F=41.53, *P*<0.001 using two-way ANOVA). In contrast, hardly any effect on the HCN current was observed in P14 mice (Figure 4D and 4F, inhibition of propofol on HCN current: −440.85 ± 55.00 pA to −425.63 ± 62.00 pA (4.65% ± 1.62%) for P14 mice, n=10, *P*>0.05 using two-way ANOVA). The effects of ketamine on HCN current were similar to those of propofol (Figure 4E and 4G, inhibition of ketamine on maximal current: −393.34 ± 61.27 pA to −379.85 ± 54.07 pA (1.2% ± 0.70%) for P14 mice, n=10, *P*>0.05 using one-way ANOVA; −672.98 ± 19.01 pA to −599.15 ± 22.19 pA (13.78% ± 2.64%) for P21 mice; −743.96 ± 23.26 pA to −613.67 ± 27.00 pA (19.99% ± 1.75%) for P28 mice; and −1165.30 ± 38.24 pA to −869.06 ± 55.35 pA (26.44% ± 7.33%) for P35 mice, n=10, F=56.16, *P*<0.001 using two-way ANOVA, exact data were shown in Supplementary Table). These results suggested that the inhibition of propofol and ketamine on HCN current increased with the development of mice, which indicated that the properties of HCN channels and HCN current may be affected by developmental processes, as the inhibiting effects of propofol and ketamine on HCN current were different.

### Age-related changes in the subunit composition of HCN channels in pyramidal neurons may contribute to the different effects of propofol and ketamine on HCN current in developmental mice

**Figure 5:**
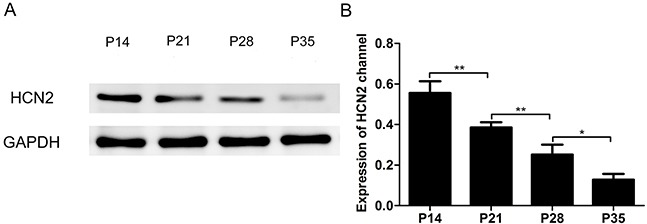
Expression of the HCN2 channel in the cortex of P14, P21, P28 and P35 mice decreased with the development of mice **(A)** Representative Western blots reveal HCN2 protein expression in the cortex of P14, P21, P28 and P35 mice. **(B)** Quantitative analysis of HCN2 channel protein (relative to GAPDH). Values are expressed as the means ± SEM. These results indicated that the expression of HCN2 channels decreased with the development of mice (n=5, F=51.16, ^*^P<0.05, ^**^P<0.01 using one-way ANOVA).

HCN1 and HCN2 channels are prominently expressed in the CNS and primarily mediate the HCN current in cortical pyramidal neurons [9, 24, 25]. Additional evidence indicates that the time constant increases with the development of mice, with the time constant of HCN1 channel activation significantly faster than that of the HCN2 channel [20, 25]. We thus speculated that the subunit composition of HCN channels in pyramidal neurons might affect the properties of the HCN current during development periods. To this end, we examined the expression of HCN channels in the cortex and observed that the expression of HCN2 channels significantly decreased with the development of mice (Figure 5, n=5, F=56.16, *P*<0.001 using one-way ANOVA), while HCN1 expression increased with the development of mice (Figure 3A and 3B). The combined expression changes of HCN1 and HCN2 channels in the cortex suggest that the increased expression of HCN1 channels and the decreased expression of HCN2 channels might contribute to the different effects of intravenous anesthetics on HCN current. These results provide primary evidence for age-related changes in the subunit composition of HCN channels.

## DISCUSSION

In the present study, we report that a single ion channel, HCN1, in the neocortex affects the sensitivity to the hypnotic effects of propofol and ketamine in mice during different postnatal developmental periods, elucidating the need for decreased anesthetics with developmental age in clinical anesthesia as discussed in previous studies [1–3]. These results further support the previous proposition that HCN1 subunits represent relevant molecular targets of propofol and ketamine anesthesia. The results of the present study can be summarized as follows. First, the LORR EC_50_ of propofol and ketamine was reduced in mice during different postnatal periods, and these behavioral results confirmed that postnatal developmental periods might be relevant for the sensitivity to the hypnotic actions of propofol and ketamine. Second, the expression of HCN1 channels increased with the development of mice and was closely correlated with the decreased LORR EC_50_ of propofol and ketamine. Thus, these data are consistent with the hypothesis that HCN1 channels play an important role in sensitivity to intravenous anesthetics in mice.

As described in previous studies, the induction dose of intravenous anesthetics in both patients and animals is influenced by age, with young animals and patients showing reduced sensitivity to anesthetics [2, 3]. In the present study, LORR analysis was performed to ascertain different responses to intravenous anesthetics in mice during different developmental periods. The results indicated that the sensitivity to the hypnotic actions of propofol and ketamine increased with the development of mice, consistent with previous studies [1–3]. A study also reported the sex differences in sodium pentobarbital maintaining anesthesia, which induced by isoflurane in rats, and ascribed the lean body mass and metabolism differences between male and female rats to the sex differences in anesthesia of sodium pentobarbital [22]. However, as our knowledge, there was no direct evidence referred to the sex differences in propofol- or ketamine- induced anesthesia in young mice. In addition, we analyzed the present data and found that there was no difference in LORR EC_50_ of propofol and ketamine in young male and female mice.

Most relevant studies typically attribute this age-related sensitivity of intravenous anesthetics to different pharmacokinetics parameters, which include the following: (1) the more rapid metabolic clearance of intravenous anesthetics in children, (2) the higher distribution volume of intravenous anesthetics in children compared with that in adults, and (3) the reduced pharmacokinetic parameters of intravenous anesthetics in the elderly [3]. However, other studies have demonstrated that the distribution volume and clearance of anesthetics are influenced by factors such as the method of administration, infusion rate, and patient covariates, and the concentration of anesthetics could not be predicted based only on pharmacokinetics parameters [25]. We thus considered that differences in the pharmacokinetics parameters of anesthetics could not deal with the observed age-related sensitivity to intravenous anesthetics and that additional factors should be investigated.

Previous studies have identified HCN1 channels as a molecular substrate of general anesthetics. Specifically, propofol and ketamine remained capable of producing anesthesia in HCN1-knockout mice at increased concentrations [9–12]. These observations indicate that HCN1 channels are relevant for the pharmacodynamics of anesthetics. Consistently, the present study confirmed that the dose requirement of propofol and ketamine anesthesia decreased with the increased expression of HCN1 channels in the cortex of mice and that the effects of propofol and ketamine on HCN current increased with development [26, 27]. Furthermore, we measured the CSF concentrations of propofol and ketamine, which directly reflect the sensitivity of the brain to these anesthetics [3], and we observed that lower levels were needed to produce the same hypnotic actions in older mice. Thus, the accumulated evidence favors the idea that HCN1 channel expression contributes to the different pharmacodynamics of anesthetics.

In the present study, we showed the increased expression of HCN1 channels and HCN current correlated with the increased sensitivity to the hypnotic actions of propofol and ketamine, and we also found the inhibition ratio of propofol and ketamine on total HCN current increased stably with the development of mice (Figure 4), while the exact reason maybe more complicated. We confer that the decreased expression of HCN2 channels may contribute to the increased inhibition ratio since there are also effects of propofol and ketamine, though weak, on HCN2 channel, indicating that the subunit composition of HCN channels also changed in developmental mice, consistent with other studies [17, 20]. *In vivo* study has demonstrated that binding kinetics of propofol to HCN channels was determined by the subunits composition and indicated less expression of insensitive HCN2 and HCN4 subunit increase affinity of propofol to HCN channels [28]. We concluded that decreased expression of HCN2 channels in cortex in our study might increase the affinity of propfol and ketamine to HCN channels but there was no evidence regarding to this binding kinetics of propofol and ketamine in old and young animals, which is under determined in future study.

Although propofol and ketamine still produced hypnosis in P14 mice, the expression of the HCN1 channel was low, and few effects of propofol and ketamine on HCN current were observed. These findings indicate that other molecular substrates might be involved in the hypnotic actions of propofol and ketamine [25–27, 29]. For instance, propofol and ketamine could modulate other receptors, such as GABAA and NMDA receptors, and these effects might also contribute to the hypnosis of propofol and ketamine, despite the expression of HCN channels in mice [25, 27].

Some limitations of the present study should be discussed. First, we validated our hypothesis only by pharmacological methods, the use of HCN knockout mice would be better way examine this hypothesis, which would be our further work. Second, we only examined the relationship between the sensitivity to propofol and ketamine and HCN channels; other anesthetics and ion channel or receptor other than HCN channels should be examined to further generalize the conclusions drawn herein. Third, we only investigated the effects of propofol and ketamine on the cortical pyramidal neurons; other brain regions such as thalamus should be evaluated in future study.

In conclusion, these results suggest that HCN channels contribute to the hypnotic actions of propofol and ketamine, as demonstrated in previous studies, and implicate the age-related developmental of HCN channels in sensitivity to the hypnotic actions of propofol and ketamine.

## MATERIALS AND METHODS

### Animals

All protocols were approved by the Institutional Animal Experimental Ethics Committee of Huazhong University of Science and Technology. The experiments were performed on wild-type C57BL/6 mice of both sexs at postnatal days 14 (P14, 6.2g ± 0.78g), 21 (P21, 10.3g ± 0.85g), 28 (P28, 14.6g ± 1.05g) and 35 (P35, 17.9g ± 1.2g). All mice were maintained under standard housing conditions with a 12-h/12-h light-dark cycle and food and drink provided ad libitum.

### Analysis of anesthetic action in mice

Bolus injections of propofol (Fresenius Kabi AB, Germany, 2.5-20 mg/kg) and ketamine (Hengrui company, Jiangsu, China, 2.5-20 mg/kg) were injected via the tail vain of each mouse (P14 to P35) as previous study described [30]. Concentrations of propofol and ketamine were diluted to 4 levels (20 μg/μl, 10 μg/μl, 5 μg/μl, 2.5 μg/μl) in order to ensure the volume of drugs are similar in different mice and exclude the effects of drugs volume on the hypnotic action of propofol and ketamine. Mice were placed in a flexible plexiglass restrain chamber and exposed the tail outside the chamber. To dilate the tail vain, moderate hot water (40°C) was used to heat the tail for 1 minute and avoid scalded. Then the tail was fixed by left hand and drug injected with right hand by a 32G insulin syringe (0.23mm × 4mm, BD Becton, Dickinson and Company, USA). Mice with failed injection were excluded. Each animal was administered a single dose of the drug on any given day and the time for administration was at 8 to 12 o’clock in the morning. Some animals received multiple concentrations (on different days, separated by at least 5 days), but no single animal contributed more than one data point for any given concentration.

Hypnosis was established as a loss-of-righting reflex (LORR) and a scale of righting reflex. We defined mice rapid forceful righting within 10 s and when placed on side as a negative LORR while no righting attempts within 10 s after completion of the injection and that persisted for at least10 s thereafter as a positive LORR [9, 10, 21].

### High-performance liquid chromatography

Cerebrospinal fluid (CSF) concentrations of propofol and ketamine in mice (P14 to P35) were determined using a slightly modified version of a previously described high-performance liquid chromatography (HPLC) method [3, 31, 32]. Mice sacrificed for cerebrospinal fluid (5 μl from foramina magnum) extraction after the injection of propofol and ketamine via the tail vein using a dose of LORR EC_50_ from the behavioral tests. One hundred microliters of acetonitrile were added to 5 μl CSF. After mixing for 1 min, the mixture was centrifuged at 2000g for 10 min at 15°C. Then, 25 μl of the supernatant was injected into the column.

Stock solutions of propofol (Sigma Chemicals Co., St Louis, MO, USA, 2000 μg/ml) were prepared in methanol. The internal standard solution was diluted with methanol-water (63:35, v/v) to 1000 μg/mL and standard solutions were prepared by further dilution of stock solutions with methanol-water (65:35, v/v). All solutions were stored at −20°C. For calibration curves, blank CSF samples (5 μl) were spiked with 10 μL of the appropriately diluted standard solutions and 10 μl of the internal standard working solution to produce final concentrations of 10, 20, 40, 80, 160 and 320 μg/ml for propofol [31].

Stock solutions of ketamine (Sigma Chemicals Co., St Louis, MO, USA, 1,000 μg/ml) were prepared in methanol. The internal standard solution was diluted with methanol-water (50:50, v/v) to 500μg/ml, and standard solutions were prepared by further dilution of stock solutions with methanol-water (50:50, v/v). All solutions were stored at −20°C. For calibration curves, blank CSF samples (5 μl) were spiked with 10 μl of the appropriately diluted standard solutions and 10 μl of the internal standard working solution to produce final concentrations of 5, 10, 20, 40, 80, and 160 μg/ml for ketamine [32].

The HPLC apparatus comprised a 501-solvent delivery system (Waters ASSOCM, Milford, MA, USA) programmed to deliver a flow of 5 μl/min using WISP 700 automatic sample injectors (Waters Assoc.) and a 486-ultraviolet detector (Waters Assoc.). The detector was used at 276 nm, and the signals were recorded and analyzed using a personal computer with Maxima 820 (Waters Assoc.) chromatography software system. An Spherisorb C_18_ reversed-phase column (5 pm particle size, 100 × 4.0 mm) was used at ambient temperature. The column was isocratically eluted with an acetonitrile-water-methanol-trifluoroacetic acid (80:20:5:0.1, v/v) solution [31, 32].

### Western blot analysis for HCN expression

To detect the expression of HCN1 channels in cortex in developmental mice, the cortex tissues from 4 groups of mice (P14 to P35) were homogenized, and the protein concentrations of the supernatant were determined using a commercial BCA kit (Pierce, Rockford, IL, USA). The supernatant (protein at 20 μg) was separated using sodium dodecyl sulfate polyacrylamide gel electrophoresis and transferred to a nitrocellulose membrane. Primary antibodies against HCN1 (#ab65706, Abcam, Cambridge, MA, USA), HCN2 (#ab65707, Abcam, Cambridge, MA, USA) and GAPDH (#ab8245, Abcam, Cambridge, MA, USA) were used. The membrane was incubated with horseradish peroxidase-conjugated anti-rabbit secondary antibody (1:10,000; Pierce) for 1 h, and the blot was developed using a Super Signal chemiluminescence detection kit (Pierce). Immunoblotting was visualized using a Kodak X-ray Processor 102 (Eastman Kodak, Rochester, NY, USA), and subsequent analysis was performed using Quantity One software (Bio-Rad Laboratories, Hercules, CA, USA) [10, 13].

### Electrophysiological recordings from mouse cortical pyramidal neurons

The mice (P14 to P35) were anesthetized (ketamine, 200 mg/kg; xylazine, 14 mg/kg, intramuscular injection), the brain was rapidly removed from the cranium and submerged in an ice-cold substituted Ringer solution bubbled with 95% O_2_ −5% CO_2_. The substituted Ringer solution contained sucrose 260 mM, KCl 3 mM, MgCl_2_ 5 mM, CaCl_2_ 1 mM, NaH_2_PO_4_ 1.25 mM, NaHCO3, 26 mM, glucose 10 mM, and kynurenic acid 1 mM. Slices (300 μm) were cut using a microslicer (DSK 1500E; Dosaka, Tokyo, Japan). Before recording, slices were incubated at 37°C for 1 h and then subsequently at room temperature in a normal Ringer solution NaCl 130 mM, KCl 3 mM, MgCl_2_ 2 mM, CaCl_2_ 2 mM, NaH_2_PO_4_ 1.25 mM, NaHCO_3_ 26 mM, and glucose 10 mM. This solution was bubbled with 95% O_2_-5% CO_2_. The slices were submerged in a recording chamber on an Olympus infrared microscope (Olympus, BX51W1, Japan), and pyramidal cells in cortex were targeted for recording based on their location in the slice and characteristic size and shape. For whole-cell voltage clamp recording of HCN channel currents, pipettes (3.5 to 5 MΩ) were filled with KCl 131 mM, NaCl 4 mM, MgCl_2_ 1 mM, CaCl_2_ 0.5 mM, HEPES 10 mM, EGTA 8 mM, MgATP 4 mM, and GTP-Tris 0.3 mM (pH 7.35~7.45, osmolarity 310 to 320 mOsmol/L). The standard bath solution contained NaCl 140 mM, KCl 3 mM, HEPES 10 mM, CaCl_2_ 2 mM, MgCl_2_ 2 mM, and glucose 10 mM (pH 7.4, osmolarity 300 to 310 mOsmol/L). Tetrodotoxin (0.5 μM; Alomone Labs, Jerusalem, Israel) was routinely added to the bath solution to inhibit action potential. Glutamate receptors were blocked with CNQX (10 μM; Sigma) and APV (50 μM; Sigma) when investigated the effects of ketamine on HCN currents. Series resistance (Rs, typically< 20MΩ) was compensated using amplifier circuits. Recordings were obtained using an Axopatch 700B amplifier (Axon Instruments, Inc., Sunnyvale, CA) at room temperature (approximately 25°C). HCN channel currents in cortical pyramid neurons were recorded as a series of hyperpolarizing step voltage pulses (−50 to −130 mV with 10 mV increments) from a holding potential of −50 mV, followed by a voltage step of −100 mV to record tail currents. The infusion rate of propofol and ketamine was 3~5 ml/min and concentration of propofol (5 μM) and ketamine (20 μM) was indicated as previous studies [9, 10, 12].

### Statistical analysis

All statistical analyses were performed using GraphPad prism 5.0 (GraphPad Software, San Diego, CA). The results are presented as the means ± SEM. Data were statistically analyzed using one-way analysis of variance (ANOVA). The effects of propofol and ketamine on HCN current were analyzed using two-way ANOVA. Dose-response data were fitted and statistically analyzed using Prism5.0 with a logistic equation with two parameters (slope and EC_50_) and affixed origin. Logistic regression was used to evaluate the correlation between the expression of HCN1 channels and HCN currents, and the CSF concentrations of propofol or ketamine. Differences in mean values were considered significant at *P*<0.05.

## SUPPLEMENTARY MATERIALS TABLE




