# Supplementary material for: HCN channels contribute to the sensitivity of intravenous anesthetics in developmental mice
Source: Oncotarget. 2018 Feb 5;9(16):12907–17. doi: 10.18632/oncotarget.24408 (PMC5849183; doi:10.18632/oncotarget.24408)
Supplement: Supplementary file 1 [file oncotarget-09-12907-s001.pdf]

HCN channels contribute to the sensitivity of intravenous anesthetics in developmental mice

SUPPLEMENTARY MATERIALS

Supplementary Table: The effect of propofol and ketamine on HCN current, Inhibition% was calculated by (I control-I anesthetic) / I control.

See Supplementary File 1
